# Disruption of the maxi-K-caveolin-1 interaction alters current expression in human myometrial cells

**DOI:** 10.1186/1477-7827-7-131

**Published:** 2009-11-23

**Authors:** Adam M Brainard, Victoria P Korovkina, Sarah K England

**Affiliations:** 1 Department of Molecular Physiology and Biophysics, Carver College of Medicine, University of Iowa, Iowa City, IA, USA; 2 Department of Obstetrics and Gynecology, Carver College of Medicine, University of Iowa, Iowa City, IA, USA

## Abstract

**Background:**

One determinant of the total K+ myometrial smooth muscle cell (MSMC) current is the large conductance, calcium- and voltage-activated potassium channel (maxi-K channel). This channel provides a repolarizing current in response to excitatory stimuli, most notably in response to increases in the levels of intracellular Ca2+, and blocking the channel by pharmacological means induces the depolarization of MSMCs and also enhances contraction strength. In MSMCs, maxi-K channels can reside in the caveolae, where they associate with the scaffolding protein caveolin-1 (cav-1). The aim of this study was to investigate the consequences of this interaction - more specifically, how disruption of the association between the maxi-K channel and cav-1 may influence the current expression and excitability of myometrial cells - with the aim of better understanding the mechanisms that underlie the regulation of normal and aberrant uterine function.

**Methods:**

Myometrial biopsies were collected from women undergoing elective C-sections. From these samples, myometrial cells were isolated, cultured, infected with a virus containing either caveolin-1 (cav-1) siRNA or scrambled cav-1 siRNA, and finally subjected to patch-clamp analysis. Mutant caveolin-binding site maxi-K channel constructs were generated and transfected into mouse Ltk- fibroblasts. Channel activity, expression, association, and localization were examined by patch-clamping, Western blot, immunoprecipitation, and immunofluorescence, respectively.

**Results:**

The caveolin-1 siRNA suppressed the total K+ current in human myometrial smooth muscle cells (hMSMC), as evident from comparison to the currents generated by both non-infected cells and cells infected with scrambled siRNA controls. The interaction between the maxi-K channel and caveolin depends on a region in the channel's C-terminal caveolin-binding site. Mutations of aromatic residues in this site (mutant F1012A, mutant Y1007A, F1012A and mutant Y1007A, F1012A, Y1015A) resulted in a decrease in K+ current compared to that produced by wild-type channels transfected into mouse Ltk- fibroblasts. However, mutation of all three aromatic amino acids (mutant Y1007A, F1012A, Y1015A) was necessary to disrupt the association between caveolin and the maxi-K channel, as visualized by immunofluorescence and immunoprecipitation.

**Conclusion:**

Our results suggest that disruption of the caveolin-binding site interferes with the cav-1/maxi-K channel interaction, and that lack of the cav-1/maxi-K channel interaction in MSMCs attenuates the total K+ channel current of the cell.

## Background

Potassium efflux from myometrial cells results in membrane repolarization. This potassium efflux constitutes the primary ionic current responsible for maintaining resting membrane potential, and contributes significantly to uterine quiescence during pregnancy. In myometrial smooth muscle cells (MSMCs), changes in the expression or activity of K^+ ^channels can translate into inadequate repolarization, thus leading to aberrant uterine activity, and this may contribute to pathophysiological conditions such as pre-term and post-term labor. One determinant of the total K^+ ^MSMC current is the large conductance, calcium- and voltage-activated potassium channel (maxi-K channel). This channel provides a repolarizing current in response to excitatory stimuli, most notably in response to increases in the levels of intracellular Ca^2+^[1], and blocking the channel by pharmacological means induces the depolarization of MSMCs and also enhances contraction strength [2]. Various mechanisms contribute to the modulation of maxi-K current expression in MSMCs. For example, an association of the channel with accessory beta subunits promotes channel activity [3]. Also, both alternative splicing of a pre-mRNA [4] and post-translational modifications of protein can lead to either increased or decreased channel activity [5]. Adding to the complexity of the regulation of MSMC excitability is recent evidence indicating that the maxi-K channel is targeted to caveolae, where it regulates cellular processes and muscle contraction [6-8].

Localization to caveolae and lipid rafts has been implicated as a regulatory mechanism for a number of ion channels. For example, isoform 4 of the cyclic nucleotide-gated channel (HCN4) has been shown to localize to lipid rafts, and disruption of this association following the application of methyl-beta-cyclodextrin results in both channel redistribution within the membrane and changes in channel kinetics [9]. In the case of the voltage-gated K^+ ^(Kv) channel, different isoforms are normally present in distinct raft domains, with Kv1.5 present in caveolae and Kv2.1 present in non-caveolar lipid rafts [10,11]. It has also been shown that cells transfected with a caveolin mutant that disrupts trafficking sequesters Kv1.5, but not Kv2.1, intracellularly. In addition, depletion of cholesterol, a key component of lipid rafts, alters Kv1.5 channel function [11,12].

Caveolar invaginations are prevalent in human MSMCs (hMSMCs), increasing the geometric cell surface area by as much as ~ 70% [13]. Maxi-K channels can reside in the caveolae, where they associate with the scaffolding protein caveolin [7,8]. Of three caveolin isoforms (cav-1, -2, and -3), cav-1 and cav-2 are predominant in both non-pregnant and pregnant non-laboring myometrium [7]. These proteins can bind, organize, and functionally regulate multiple cell signaling molecules [11] through a region termed the scaffolding domain, which interacts with a variety of proteins, including G-protein alpha-subunits, Src family tyrosine kinases, and eNOS [14,15].

In spite of our knowledge of the existence of an association between the maxi-K channel and caveolin, how this interaction affects MSMC function remains unknown. In this study we investigate the consequences of this interaction--more specifically, how the association between the maxi-K channel and cav-1 influences the current expression and excitability of myometrial cells--with the aim of better understanding the mechanisms that underlie the regulation of normal and aberrant uterine function.

## Methods

### Generation of mutants

Constructs encoding mutant maxi-K channel forms were generated using the QuikChange Site-Directed Mutagenesis Kit (Stratagene, La Jolla, CA). Primers used are as follows:

a) Mutant F1012A,

Forward 5'-GGTATAATATGCTTTGTGCTGGAATTTACCGGCTGAGAGATGCG-3', Reverse 5' CGCATCTCTCAGCCGGTAAATTCCAGCACAAAGCATATTATAGG-3';

b) Mutant Y1007, F1012A,

Forward 5'-GCA AAGCTCTGAAAACAGCTAATATGCTTTGTGCTGG-3',

Reverse 5'-CCAGCACAAAGCATATTAGCTGTTTTCAGAGCTTTGC-3'; and

c) Mutant Y1007A, F1015A, Y1015A,

Forward 5'-CTTTGTGCTGGAATTGCCCGGCTGAGAGATGCGCACCTC-3',

Reverse 5'-GAGGTGCGCATCTCTCAGCCGGGCAATTCCAGCACAAAG-3'.

For both patch-clamping and immunocytochemistry experiments, mutants were cloned into the pTracer expression vector, (Invitrogen, Carlsbad, CA) between restriction sites Not1 and Xba1.

### Tissue collection

Human myometrial tissue from the lower uterine segment was obtained from patients who, in the absence of spontaneous or induced labor contractions, underwent elective Cesarean section in late pregnancy (NL; 38-40 wk gestation) while under spinal anesthesia. All patients signed written consent forms approved by the University of Iowa's Internal Review Board (approval no. 199809066). Tissue was placed in Hanks' balanced salt solution or in phosphate-buffered saline (PBS) on ice, and was used to isolate hMSMCs within 1-3 h of collection.

### Isolation, culture, transfection, and infection of cells

Human MSMCs were isolated and cultured as previously described [7], and were then utilized to study the effects of caveolins on endogenous cells by patch clamp analysis The adenoviral constructs containing caveolin-1 and caveolin-1-scrambled siRNAs were a gift from Dr. Debbie Thurmond (Indiana University School of Medicine), and their production was previously described [16] (Viraquest, North Liberty, IA). Cells were infected with 1 μl of 1.0 - 1.1 × 10^12 ^particles/ml of the purified siRNA adenoviral constructs and incubated in the presence of the virus for an additional 72 hours before experiments were carried out. Transduction efficiency was gauged by EGFP fluorescence every 24 hours, which was typically greater than 90% by 72 hours.

The mouse Ltk- fibroblasts (Ltk-) [17] used for patch clamp, Western blot, immunoprecipitation and immunofluorescence studies were generated by transfecting cells of the Ltk- parent line, following growth to 50-80% confluency in T-75 flasks, 6-well or 12-well dishes, in DMEM/F12 (Sigma, St. Louis, MO) supplemented with 10% FBS (Gibco-BRL, Carlsbad, CA) and 50 μg/ml gentamicin (Sigma). Ltk- cells were transfected with 10 μg (T-75), 2 μg (6-well) or 1 μg (12-well) of plasmid DNA (LipofectAMINE PLUS Reagent Kit, Invitrogen), and incubated an additional 48 hours before the experiments were carried out. The Ltk- cells utilized for these experiments lack endogenous maxi-K channels but contain cav-1; this enabled us to study each caveolin-binding mutant without contamination from the endogenous channel.

### Preparation of lipid rafts

Ltk- cells transfected with plasmid DNA (one T-75 flask) were trypsinized (0.25% TE/EDTA, Gibco-BRL) and spun down at 300 × g for 5 min. Cells were resuspended in 200 μl of Mes-buffered saline (MBS; 24 mM Mes, 150 mM NaCl, pH 6.5) plus 1% Triton X-100 and a Complete Protease Inhibitor tablet (Roche, Indianapolis, IN), incubated for 30 min on ice and dounce homogenized on ice for 1 min. A 100 μl aliquot was transferred to a microcentrifuge tube, and 300 μl of 53.33% sucrose/MBS was added and mixed in. 450 μl each of 30% and 5% sucrose/MBS were layered above this mixture, in this order, and the samples were centrifuged for 24 hours at 54,000 rpm, at 4°C. After this spin, 10 equal fractions were collected, starting at the top. We controlled for the presence of non-lipid membranes by monitoring the presence of the human transferrin receptor by Western blot, as this protein does not associate with lipid rafts.

### Preparation of beads and immunoprecipitation

Protein G-Plus Agarose beads (Santa Cruz, Santa Cruz, CA) were incubated in 4 ml Dulbecco's Phosphate Buffered Saline, DPBS, (Gibco-BRL) with 1% BSA, 1% Normal Donkey Serum and 1% Normal Rabbit Serum, for 2 hours at 4°C. The beads were washed 1× with DPBS and resuspended in 1 ml DPBS with 0.025% Sodium Azide. 50 μl of each floating fraction were incubated in 1 ml Solubilization Buffer (10 mM Tris pH 8.0, 150 mM NaCl, 1% TX-100, 60 mM Octyl-glucopyranoside) plus a Complete Protease Inhibitor Tablet (Roche), for 45 min at 4°C, and were then mixed by nutation. These samples were spun down at 3500 × g for 5 min at 4°C, and the supernatants were transferred to new tubes. 50 μl of the prepared beads were added, and the samples were agitated overnight at 4°C. One μg of a maxi-K channel antibody (BD Biosciences, San Jose, CA) was then added, and immunoprecipitates were gently mixed overnight at 4°C. The samples were spun down at 3500 × g at 4°C, and washed 3× with 1 ml Buffer A (150 mM NaCl, 50 mM Tris-HCl pH 7.5, 1 mM EDTA, 0.5% TX-100), after which the bead fractions (representing the immunoprecipitates) were resuspended in 40 μl of 2× sample loading buffer (4X: 10% Glycerol, 0.25 M Tris pH 7.0, 3% SDS, 5% 2-Mercaptoethanol, Bromophenol Blue).

### Immunoblotting

The immunoprecipitates and mini lipid-raft fractions were separated by SDS-PAGE and immunoblotted as previously described [4]. The primary antibodies used were one against the mouse maxi-K channel (1:250) (BD Biosciences), one against mouse cav-1 (1:1000) (BD Biosciences) and one against the mouse transferrin receptor (1:1000) (Zymed, South San Francisco, CA). The secondary antibody used in all cases was goat anti-mouse (1:3000) (Jackson Immunoresearch, West Grove, PA). All antibodies were diluted in PBST (1.37 mM NaCl, 81.01 mM Na_2_HPO_4_, 26.82 mM KCl, and 14.7 mM KH2PO4, 0.5% Tween, pH 7.2) containing 3% non-fat dry milk. Immunoreactivity was detected using HyGlo Western Blotting detection reagents (Denville Scientific Inc., Metuchen, NJ).

### Immunocytochemistry

Ltk- cells transfected with plasmid DNA were fixed with 2% paraformaldehyde for 30 min at RT, quenched with 50 mM NH_4_Cl for 10 min at RT, permeabilized with 0.1% Triton X-100 for 10 min at RT, and blocked with 10% heat-inactivated FBS + 1% heat-inactivated donkey serum (blocking buffer) for 30 min at 37°C (all diluted in DPBS). For co-localization studies, Ltk- cells were incubated with rabbit maxi-K channel antibody (1:250; Millipore, Temecula, CA) for 1 hour at 37°C, and then with a Cy3-conjugated donkey anti-rabbit (1:1000; Jackson Immunoresearch) for 15 min, 37°C. They were then incubated for 15 min in blocking buffer for at 37°C, for 30 min in blocking buffer plus a mouse anti-caveolin antibody (1:500; BD Biosciences) for 30 min at 37°C, and finally for 15 min in blocking buffer plus a Cy5-conjugated donkey anti-mouse (1:1000; Jackson Immunoresearch) for 15 min at 37°C. Signals were visualized using a Zeiss 510 confocal scanning microscope (Zeiss, Oberkochen, Germany), and images were acquired with a Zeiss LSM 5 Image Browser (Zeiss). For controls, Ltk- cells were incubated with primary antibodies alone (30 min at 37°C) or secondary antibodies alone (15 min at 37°C). In general, Ltk- cells do not generate high background when an anti-mouse secondary antibody is used. Moreover, any background issues due to non-specific binding of the secondary antibody would be discovered by performing the experiments with the secondary antibody alone. Bleedthrough of fluorescent signals into the neighboring channels was controlled for by turning off all but one laser at a time, and recording images in all channels. Laser power, pinhole size and detector gain were then adjusted to ensure that the image appeared in its respective channel only. A nuclear counterstain was not performed since confocal microscopy of these cells showed sufficient morphological detail to identify individual cells.

### Electrophysiology

All patch-clamping experiments were performed at RT. Whole-cell current was measured using an Axopatch 200-B amplifier (Molecular Devices, Sunnyvale, CA). Signals were filtered with a cutoff frequency of 5 kHz. Data acquisition was controlled using commercial pClamp 9.2 software (Molecular Devices), and data were digitized using a Digidata 1320 interface (Molecular Devices). Ltk- cells expressing wild-type and mutant channels (see Transfections), and hMSMC in which the caveolin-1 gene was inhibited (see siRNA) were placed in a pH 7.4 bath solution containing, in mM: 135 NaCl, 4.7 KCl, 1 MgCl_2_, 10 Glucose, 2 CaCl_2 _and 5 Hepes. Borosilicate glass pipettes of 2-5 MΩ were filled with a pH 7.2 solution containing, in mM: 140 KCl, 0.5 MgCl2, 1 EGTA, 5 ATP and 5 Hepes. Cells expressing GFP (the reporter for the plasmid and siRNA expression vector used) were clamped, currents were measured using a holding potential of -80 mV and prepulsing to -100 mV, and currents were elicited at step potentials from -80 to 160 mV in 20-mV intervals.

### Statistical analysis

Each experiment was performed in a minimum of X independently transfected/infected samples. Statistical significance was calculated using Student's *t*-test for unpaired observations (siRNA and caveolin binding-site mutation experiments) and paired observations (for IbTX experiments; SigmaPlot software; SPSS, Chicago, IL). Differences were considered significant at *P *< 0.05.

## Results

### The maxi-K channel-generated current in human MSMCs is reduced when caveolin-1 is knocked down

**Figure 1:**
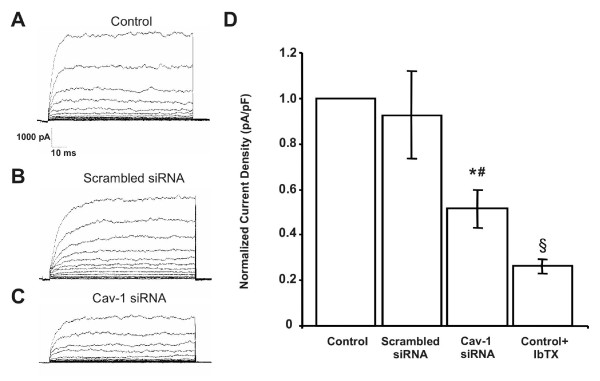
**Whole-cell patch-clamp analysis of hMSMCs depleted of caveolin-1 using an siRNA approach**. siRNA-mediated depletion of caveolin-1 (cav-1) in non-laboring hMSMCs (C) decreases current density relative to that in non-infected hMSMCs (A) and hMSMCs treated with a scrambled siRNA (B). (D) Bar graph summarizes and compares the mean current densities presented in A-C. Inhibiting cav-1 resulted in a 49% decrease in the mean current density compared to that in non-infected control cells, and a 45% decrease in comparison to that in samples treated with a scrambled siRNA. In non-infected control cells, IbTX decreased the mean current density by 74%. Significant differences (p < 0.05) between samples treated with cav-1 siRNA and non-infected controls (*) or scrambled siRNA (#) are noted. (§ indicates significant difference between non-infected controls in the presence and absence of IbTX. Data shown are the mean values for 16 (control), 6 (scrambled siRNA control), 14 (cav-1 siRNA) and 3 (control+IbTX) independent experiments ± standard error.

To establish whether interactions between maxi-K channels and caveolin affect the endogenous myometrial total outward K^+ ^current, we used a siRNA to inhibit cav-1 gene expression in hMSMCs. Maxi-K currents were measured by whole-cell patch clamping and compared to those from control (non-infected cells and cells infected with scrambled siRNA; Fig. 1A, B, and 1C). Current in cells depleted of cav-1 was decreased 49% in comparison to that in control cells (Fig. 1D), and 45% in comparison to that in cells transfected with scrambled siRNA (Fig. 1D). In control cells, iberiotoxin (IbTX) inhibited K^+ ^current by 74%. Our data suggest that a subpopulation of maxi-K channels whose function is modulated by cav-1 generates 40 to 45% of the total maxi-K current in hMSMCs. The decrease in current density when cells are depleted of cav-1 could reflect a role for cav-1 in trafficking of the maxi-K channel to the plasma membrane. The fact that the current is not abolished demonstrates that maxi-K channels are not solely modulated by cav-1.

### The maxi-K channel-generated current in mouse fibroblasts is reduced when the channel's caveolin-binding motif is disrupted

**Figure 2:**
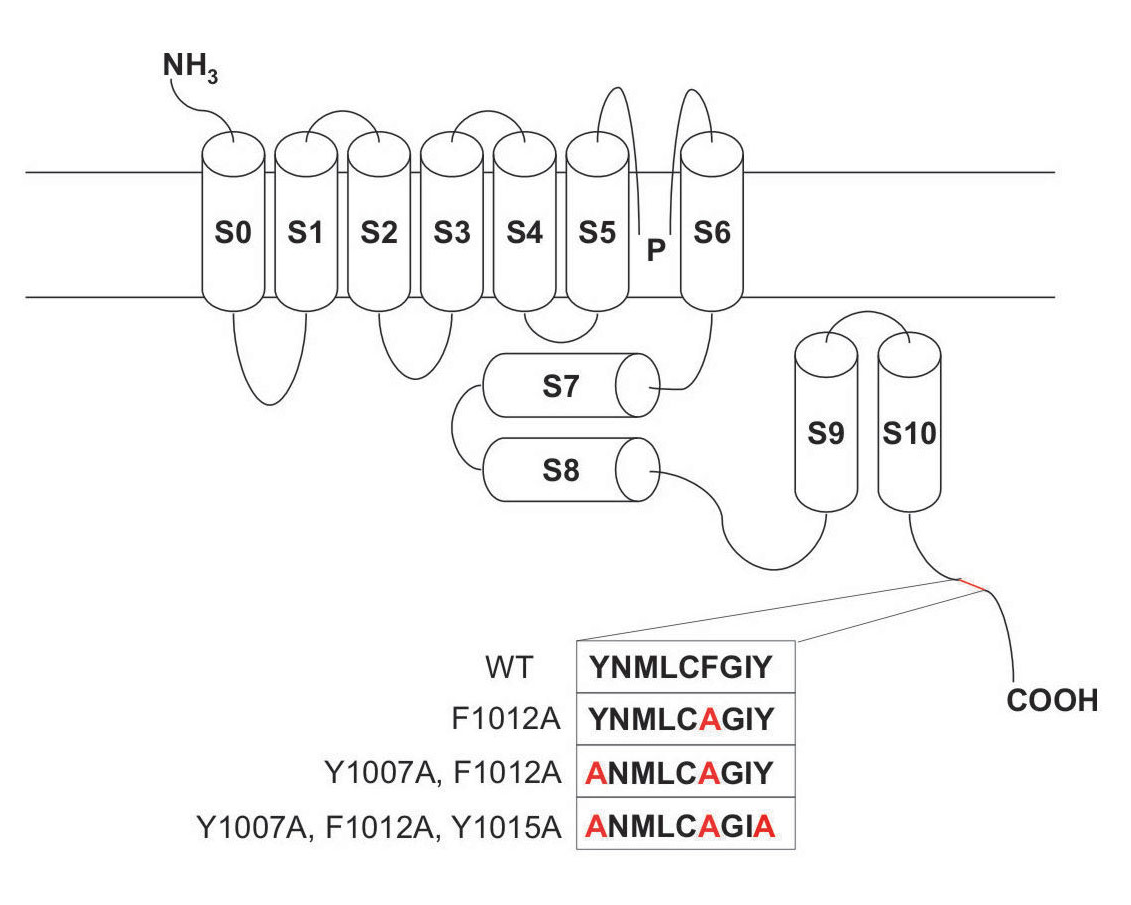
**Schematic representation of the maxi-K channel**. The channel is composed of seven hydrophobic membrane-spanning domains (S0-S6) and four intracellular hydrophobic domains (S7-S10). The caveolin-binding motif is shown in the inset, along with the amino-acid alterations present in each of the mutants tested in this study.

**Figure 3:**
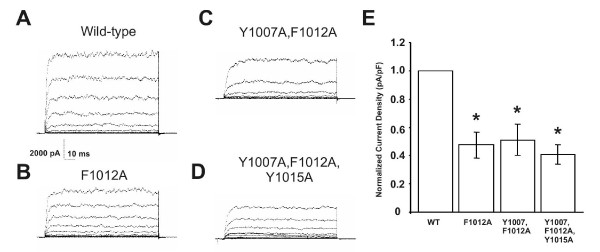
**Whole-cell patch-clamp analysis of mouse fibroblasts transfected with wild-type and mutant forms of the maxi-K channel**. Current density recordings for Ltk- cells transfected with (A) WT, (B) mutant F1012A, (C) mutant Y1007A, F1012A and (D) mutant Y1007A, F1012A, Y1015A maxi-K channel constructs. (E) Bar graph data summarizes and compares the mean current densities generated by cells transfected with the indicated maxi-K channel construct. Cells transfected with mutant F1012A, mutant Y1007A, F1012A and mutant Y1007A, F1012A, Y1015A exhibited 53%, 49% and 59% decreases in current density, respectively, in comparison to cells transfected with the WT construct. (*) indicate statistically significant differences between currents generated in cells transfected with each mutant maxi-K channel construct versus cells transfected with the WT construct (p < 0.05). Data shown are the mean values for 10 (WT, mutant F1012A, mutant Y1007A, F1012A) and 7 (mutant Y1007A, F1012A, Y1015A) independent experiments ± standard error.

The maxi-K channel contains one caveolin-binding motif in its C-terminus (^1007^YNMLCFGIY^1015^) (Fig. 2). In order to determine the role of this site in modulating maxi-K channel localization and activity, we substituted the aromatic amino acids within this motif with alanines in various combinations (Fig. 2). In order to identify the functional significance of each mutation without contaminating native current, we performed experiments in Ltk- cells, which lack endogenous maxi-K channel current but contain endogenous cav-1. Each mutant was assessed by performing whole-cell patch-clamp measurements of K^+ ^currents in Ltk- cells transiently expressing the mutants, and then comparing current expression in these mutants to that in WT controls (Fig. 3A). Mutant F1012A and mutant F1007A, Y1012A generated less current than the WT channel (53% decrease with mutant F1012A, Fig. 3B, E; 42% decrease with mutant F1007A, Y1012A, Fig. 3C, E). Mutant Y1007A, F1012A, Y1015A was associated with the most significant reduction in current density (59% decrease, Fig. 3D, E). These findings suggest that cav-1 contributes to the regulation of maxi-K current, at least in part, via specific maxi-K-caveolin interactions.

### The maxi-K channel fails to associate with lipid rafts when its caveolin-binding motif is disrupted

**Figure 4:**
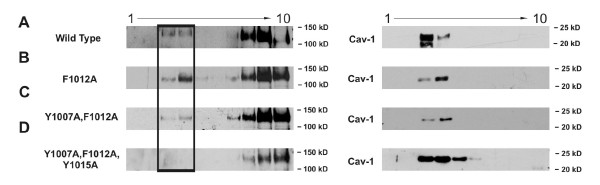
**Western immunoblot analysis of the expression of WT and mutant maxi-K channels in lipid rafts from mouse fibroblasts**. Sucrose density gradient centrifugation of 1% Triton X-100 solubilized extracts from transfected Ltk- cells were examined by Western blot analysis. Samples range from lowest density at left (lane 1) to highest density at right (lane 10). The (A) WT (B) mutant F1012A, and (C) mutant Y1007A, F1012A forms of the maxi-K channel (as indicated by boxed regions in panels on left) co-fractionate with caveolin-1 in low-density fractions (cav-1; right). However the (D) mutant Y1007A, F1012A, Y1015A form (visible at left) was not present in these low-density caveolin-associated fractions. Data shown are representative examples from 10 independent experiments for each protein form.

Since mutation of the caveolin-binding site had a significant effect on activation of the maxi-K channel, we next determined the role of the caveolin-binding motif in regulating localization of the maxi-K channel within lipid rafts. Taking advantage of the characteristics of lipid rafts--in particular their resistance to solubilization in nonionic detergents at low temperature and their buoyancy due to an enriched lipid content--we were able to isolate Triton X-100-insoluble complexes from mouse Ltk- cells transfected with wild-type (WT) and mutant maxi-K channels. Western blot analysis of lipid raft fractions using a maxi-K channel antibody confirmed that WT maxi-K channels were present in fractions that also harbored cav-1 (Fig. 4A, boxed region). Like the WT channels, the mutant channels containing either one (F1012A), or two (Y1007A, F1012A) amino-acid substitutions were present in the same fractions (Fig. 4B and 4C boxed region). However, the mutant with three amino-acid substitutions (Y1007A, F1012A, Y1015A) was absent from these fractions, with its expression limited to non-caveolar lipid rafts (Fig. 4D). The solubilization of non-raft proteins in these samples was complete, as the transferrin receptor (which does not associate with lipid rafts and thus served as a marker of Triton X-100-soluble membranes) was not detected in any of these samples (data not shown).

### Disruption of the maxi-K channel's association with caveolin-1 in mouse fibroblasts leads to channel mislocalization

**Figure 5:**
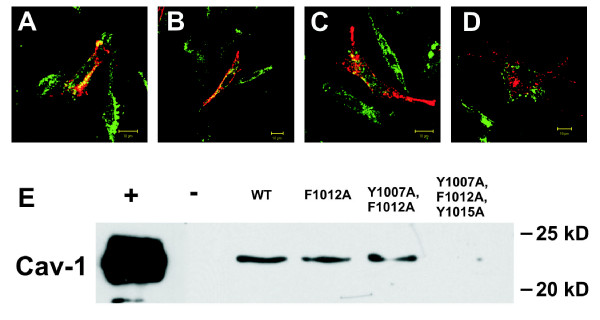
**Immunofluorence- and immunoprecipitation-based analyses of cellular localization of wild-type and mutant forms of the maxi-K channel in mouse fibroblasts**. Cellular localization of (A) WT, (B) mutant F1012A, (C) mutant Y1007A, F1012A and (D) mutant Y1007A, F1012A, Y1015A maxi-K channel forms in Ltk- cells, as detected by immunocytochemistry, using antibodies against the maxi-K channel (red) and cav-1 (green). Images were pseudocolored for better comparison. WT and all mutant channels except mutant Y1007A, F1012A, Y1015A co-localized with cav-1 on the membrane (yellow). Data shown are representative examples of 6 (WT, mutant F1012A, mutant Y1007A, F1012A) or 5 (mutant Y1007A, F1012A, Y1015A) independent experiments. (E) Immunoprecipitation of the lipid raft fractions shown in the boxed region of Figure 4 (using an anti Maxi-K channel antibody) demonstrated that WT, mutant F1012A, and mutant Y1007A, F1012A, but not mutant Y1007A, F1012A, Y1015A, associate with cav-1 (E). Data shown are representative of 8 (WT), 10 (mutant F1012A), 9 (mutant Y1007A, F1012A) and 6 (mutant Y1007A, F1012A, Y1015A) independent experiments.

Since caveolae share many biochemical properties with lipid rafts, the isolation of detergent-resistant fractions is not sufficient to discriminate between these two subcellular domains. We therefore used anti-channel and anti-caveolin antibodies in immunocytochemical analysis to determine whether, in Ltk- cells expressing the WT and mutant channels, the channel proteins and caveolin localize to the same area of the membrane. As expected, WT maxi-K channels (Fig. 5A, red) were localized primarily on the plasma membrane, whereas cav-1 (Fig. 5A, green) was present both on the plasma membrane and intracellularly. Regions of overlap between the cav-1 and WT signals (Fig. 5A, yellow) were observed in these cells, consistent with co-localization at the plasma membrane. Similarly, mutant F1012A (Fig. 5B) and mutant Y1007A, F1012A (Fig. 5C) were expressed only on the plasma membrane, in a pattern that overlapped with that of cav-1. In contrast, mutant Y1007A, F1012A, Y1015A localized to both the membrane and intracellular sites, and its signal did not overlap with that of cav-1 (Fig. 5D).

To confirm that the reduction in maxi-K current observed in cells expressing the triple mutant is due to the altered interaction between the maxi-K channel and cav-1, we immunoprecipitated the maxi-K proteins from isolated lipid rafts (as indicated by a box in Figure 4). In the case of lipid rafts isolated from cells transfected with the WT channel (Fig. 5E, WT), mutant F1012A (Fig. 5E, F1012A) and mutant Y1007A, F1012A (Fig. 5E, Y1007A, F1012A), cav-1 was observed in the immunoprecipitate. However, when lipid rafts from cells transfected with mutant Y1007A, F1012A, Y1015A were tested, cav-1 was absent (Fig. 5E, Y1007A, F1012A, Y1015A). These findings suggest that all three aromatic amino acids contribute to the interaction between the maxi-K channel and cav-1, but that two are sufficient to promote a detectable interaction.

## Discussion

The molecular mechanisms that underlie the process, and particularly the initiation, of labor remain only poorly understood. During labor, the upper segment of the uterus actively contracts, thereby promoting fetal expulsion, whereas the lower portion of the organ (composed of both the lower uterine segment and the cervix) relaxes to permit fetal exit. Cells isolated from the lower uterine segment of humans during late stages of pregnancy generate a current reminiscent of that typically produced by the maxi-K channel, but this channel becomes constitutively active and lacks voltage- and Ca^2+^-sensitivity after the onset of labor [18-20]. The reason for this switch in activity remains unknown, but could be due to: 1) variation in the physiological and pharmacological properties of maxi-K channels in MSMCs during pregnancy and/or, 2) rapid changes in maxi-K channel regulation during the transition from the non-laboring to the laboring state. Our recent findings that the maxi-K channel resides in caveolae on the MSMC, and that it associates with the actin cytoskeleton to regulate channel activity, raise the possibility that this channel may interact dynamically with associated proteins to regulate the transition of the uterus from a quiescent to a contractile state. For example, components crucial for the regulation of Ca^2+^, a key regulator of the maxi-K channel, are localized in caveolae and may serve as initiation sites for Ca^2+ ^release events, including Ca^2+ ^sparks [21,22]. These data suggest that the caveolar microdomain may play a role in modulating myometrial excitability.

Myometrial smooth muscle contains an abundance of caveolae, which have been proposed to expose or shield the associated proteins to/from the extracellular milieu by opening and closing, respectively [23]. The vast number of caveolae in the myometrium is likely a reflection of the rich signaling environment. Indeed, several studies have shown that caveolae serve as docking sites for many cell-signaling molecules involved in muscle contraction, i.e. PKCα and rhoA [24]. Moreover, hormones regulate the number of caveolae, with estrogen decreasing caveolin levels and thereby the number of caveolae, and progesterone having the opposite effect [25]. Based on these findings, we believe that maxi-K channels that are present in caveolae and interact with caveolin would likewise be responsive to the negative effects of estrogen, and that the decrease in channel current expression could account for the more depolarized myometrial membrane and enhanced contractility during labor. However the nature and role of the interaction between caveolar proteins and the maxi-K channel in the myometrium had not been identified prior to this study.

The maxi-K channel contains a consensus caveolin-binding motif (^1007^YNMLCFGIY^1015^) in its C-terminus [26], and mutation of phenylalanine within the caveolin-binding motif had previously been shown to disrupt the association between the ganglioside-specific sialidase Neu3 and cav-1 [27]. Here we show that although mutation of the aromatic amino acids that constitute a part of this motif leads to diminished current regardless of the number and placement of substitutions, only the triple-mutant Y1007A, F1012A, Y1015A loses its ability to associate with cav-1. A recent study investigating similar maxi-K channel mutants in HEK 293 cells found that single, double and triple substitutions of these aromatic amino acids fail to disrupt the interaction between the maxi-K channel and cav-1, but that complete deletion of the consensus site abolishes almost all (80-85%) interaction with cav-1 [8]. This is inconsistent with our demonstration that mutant Y1007A, F1012A, Y1015A was not present in caveolin-containing fractions, and that it neither immunoprecipitates with cav-1 or co-localizes with it at the plasma membrane. Although we cannot fully explain the differences between the results from the two studies, one possible source of this discrepancy is the fact that the HEK293-cell study relied on the use of heterologous over-expression of both the maxi-K channel proteins and cav-1 (in caveolin-negative HEK293 cells) [8]. In contrast, the Ltk- mouse fibroblasts used in our study expressed endogenous cav-1, thus providing a more physiological environment for the study of caveolin-maxi-K interactions. We stress that, in spite of the differences between the two studies, they do agree on the localization and amino acid sequence of the consensus site that mediates binding of the maxi-K channel to caveolin [8].

Inhibiting the cav-1 gene in hMSMCs demonstrated the significance of the maxi-K-caveolin interactions for the excitability of myometrial cells. The decrease in hMSMC current density observed in these experiments complements the findings of Shmygol et al., who observed that disrupting lipid rafts by depleting cells of cholesterol also decreased the maxi-K current in freshly dispersed myometrial cells [28]. The expression of caveolin isoforms and their association with maxi-K channels change during pregnancy [7]. It is thus possible that changes in the association of the maxi-K channel with various caveolin isoforms plays a role in determining which other cell signaling molecules the maxi-K channel is in proximity to during different stages of pregnancy.

Interestingly, all three of the mutants used in this study reproduced the inhibitory effects of cav-1 deficiency on maxi-K channel current in hMSMCs, although only in the case of mutant Y1007A, F1012A, Y1015A was the maxi-K-caveolin complex disrupted. These data suggest that the cav-1 regulates the maxi-K channel at a minimum of two independent steps: 1) complex formation and 2) current modulation. Formation of the maxi-K-caveolin complex may involve redundant pathways, thus making it difficult to completely disrupt this complex. In fact, Alioua et al. have identified a second putative caveolin-binding site in the maxi-K channel protein [8]. Thus, we hypothesize that extensive changes in amino acid sequence within the caveolin-binding site are required to dissociate the channel and cav-1. Given that the physical formation of the maxi-K-caveolin complex may be insufficient to regulate all maxi-K channel activity, a second mechanism involving only the ^1007^YNMLCFGIY^1015 ^motif may contribute to the ability of cav-1 to activate the maxi-K-generated current. Single amino-acid substitutions may disturb this mechanism so that cav-1 cannot maintain the maxi-K current at physiological levels. The caveolin-binding motif is located within a single exon of the KCNMA1 gene, which encodes the maxi-K channel. This raises the possibility that the heavily alternatively-spliced KCNMA1 transcript could produce a channel lacking this caveolin-binding motif--which this study and another [8] have shown to lead to a decrease in the current expressed by the channel. However, experiments seeking to identify a protein that encodes a channel without a caveolin-binding motif were inconclusive (data not shown). While it is possible that lacking this entire domain is necessary to produce physiologically relevant changes in current expression, even point mutations might be sufficient to do so. More experiments in this area are needed to convincingly resolve this issue.

The C-terminus of the maxi-K channel harbors not only the caveolin-binding motif, but also an actin-binding domain [29] and is capable of interacting with the actin-binding protein filamin [30]. Our own work has shown that the maxi-K channel associates with actin, and has suggested that an actin-channel-caveolin complex could potentially be involved in the regulation of myometrial smooth muscle K^+ ^current [7]. Further elucidation of how all of these regulatory mechanisms interact is expected to shed significant light on the regulation of myometrial contractility.

## Conclusion

Our results showing that inhibiting the cav-1 gene in hMSMCs leads to decreased maxi-K channel current demonstrate the significance of the maxi-K-caveolin interactions for the excitability of myometrial cells. These interactions appear to be regulated, in part, by the aromatic amino acids located within the caveolin-binding motif of the maxi-K channel. Because the maxi-K channel is important for regulating myometrial cell excitability and uterine contraction, the modulation of this channel by dynamic interactions with caveolin could, in part, explain novel mechanisms underlying aberrant uterine activity.

## Competing interests

The authors declare that they have no competing interests.

## Authors' contributions

AMB and VPK carried out all experimental work. AMB, VPK, and SKE conceived of the study and designed all experiments together. All authors participated in the writing and editing of the final manuscript and approved of the final version.
